# Improving the Strength of Eucalyptus Wood Joints Through Optimized Rotary Welding Conditions

**DOI:** 10.3390/ma18245596

**Published:** 2025-12-12

**Authors:** Jiankun Liang, Xiao Zhong, Yuqi Yang, Guifen Yang, Shuang Yin, Feiyan Gong, Chuchu Chen, Huali Li, Tong Meng, Yulan Jian, De Li, Caihong Long, Zhixian Song, Zhigang Wu

**Affiliations:** 1 College of Civil Engineering, Kaili University, Kaili 556011, China; dushimensheng@126.com; 2 College of Forestry, Guizhou University, Guiyang 550025, China; 18985361700@163.com (X.Z.); 15180721265@163.com (Y.Y.); 15772783270@163.com (G.Y.); yinshuang824@163.com (S.Y.); 15285111783@163.com (F.G.); 17384255170@163.com (C.C.); 17385535230@163.com (H.L.); 18188101139@163.com (T.M.); chlong0927@163.com (C.L.); szx3520388395@163.com (Z.S.); 3 School of Chemistry and Material Engineering, Zhejiang A&F University, Hangzhou 311300, China

**Keywords:** eucalyptus wood, rotary welding, microstructural analysis, thermal stability, interfacial changes, densification

## Abstract

Conventional wood connections rely on adhesives and metal fasteners, causing environmental concerns. Wood rotary welding offers an adhesive-free alternative. This study systematically investigated rotary welding of eucalyptus wood, evaluating process parameters’ effects on joint performance. Chemical and microstructural transformations at the welding interface were characterized using FT-IR, XPS, XRD, SEM, and TGA. Optimal parameters significantly enhanced connection strength compared to unwelded specimens. The welding process induced partial degradation of hemicellulose and cellulose, forming new chemical bonds and increasing carbonyl compounds. XRD revealed increased wood crystallinity, while SEM showed tighter interfaces with enhanced mechanical interlocking. TGA confirmed improved thermal stability at the welded interface. The findings demonstrate that rotary welding improves eucalyptus wood joint strength through combined chemical, thermal, and structural modifications, providing guidance for optimizing welding protocols in sustainable wood manufacturing.

## 1. Introduction

Predominant reliance on adhesives and metal fasteners in conventional wood connections introduces significant drawbacks: chemical contamination, corrosion, and barriers to material recycling [1,2,3,4,5]. These concerns have been amplified by mounting pressures for environmental sustainability. Consequently, adhesive-free bonding technologies have attracted considerable research focus. Wood welding operates through frictional heat generated between components, melting lignin and hemicellulose into a natural adhesive matrix that solidifies upon cooling [6,7,8]. This approach delivers rapid processing, eliminates synthetic adhesives and volatile emissions, and facilitates end-of-life recovery—establishing it as a promising complementary alternative to traditional joinery, mechanical fastening, and synthetic bonding [9,10]. Extensive development has been pursued by European research institutions in France, Germany, and Switzerland, with implementation advancing progressively in structural timber applications and furniture manufacturing.

The concept of wood welding was first proposed in 1996. Suthoff et al. [11] published a patent on wood friction welding, which involves connecting wood pieces solely using wood and achieving relatively good connection performance. Compared with traditional connection techniques, it is more environmentally friendly and is considered to have strong potential for application. Depending on the welding method, wood welding is mainly divided into linear friction welding and rotary tenon welding. Linear friction welding involves high-speed frictional movement of two or more wood pieces under a certain pressure. The heat generated from friction melts the components in the wood (mainly lignin and hemicellulose). After the frictional movement stops, the molten polymer cools to form a relatively stable welding interface, thereby achieving wood welding [12,13,14,15]. Rotary tenon welding involves inserting a rotating round tenon into a pre-drilled hole under a certain pressure. The outer side of the round tenon rubs against the outer side of the hole to create a molten interface, which solidifies to achieve the connection. Linear friction welding has poor water resistance under conditions of high moisture content and is prone to separation, while rotary tenon welding has stronger water resistance and better stability [16,17,18]. The tenon is often used in the furniture industry to connect parts of panel furniture. Over the years, Europe has applied rotary tenon welding technology to the connection of furniture components and has conducted relevant application research. It has been found that products produced by this technology meet the requirements for structural strength and product stability, so rotary tenon welding technology has a more promising application in furniture components and wooden building components.

Precise control of parameters is crucial for improving the performance of rotary tenon welding. Key parameters involved in rotary tenon welding include welding speed, the ratio of pre-drilled hole diameter to tenon diameter, tenon insertion angle, and tenon insertion depth [16,18,19]. Optimal parameter settings require careful balance. Rotational speed must be controlled—excessive velocity generates frictional heat that carbonizes or deforms wood fibers, while overly slow rotation extends processing time and yields weak bonds [19,20,21,22]. Pre-drilled hole dimensions relative to tenon diameter demand similar precision: oversized holes create loose fits with insufficient strength, whereas undersized holes impede insertion and risk damaging the wood. Insertion angle critically affects contact quality during welding; improper alignment reduces interface area and compromises joint integrity [4,19,23,24,25]. Penetration depth likewise requires optimization—shallow insertion limits bonding area, while excessive depth may fracture the substrate or mar surface appearance [25,26,27].

Although rotary welding has been explored across various wood species, systematic investigation of eucalyptus remains limited, particularly regarding the mechanistic relationship between process parameters and joint performance. This study bridges that gap through controlled experiments evaluating key welding conditions and characterizing the interface using multiple analytical techniques to track chemical and physical transformations. These findings provide practical guidance for optimizing eucalyptus welding protocols.

## 2. Materials and Methods

### 2.1. Materials

The experiments were conducted using the following wood species and components. Eucalyptus wood *(Eucalyptus robusta* Smith) (0.73 g/cm^3^, 12% moisture), sourced from Rongjiang County, Guizhou, China (109°21′ E, 26°24′ N), with a tree age of approximately 15 years. Schima wood (*Schima superba* Gardn) tenons (0.67 g/cm^3^), Zelkova wood (*Zelkova serrata* (Thunb.) Makino) tenons (0.64 g/cm^3^), and Eucalyptus wood tenons (0.73 g/cm^3^) all at 12% moisture were used in the experiments [19]. All tenons had a diameter of 10 mm and a length of 80 mm.

### 2.2. Pretreatment of Eucalyptus Wood Substrate

Eucalyptus wood was visually selected based on uniform color and absence of discoloration, blackening, white spots, knots, and decayed sections. The texture was inspected to ensure clear, natural grain without twisting or breakage. After cleaning and drying the surface, the wood was cut into 50 mm × 40 mm × 30 mm specimens using a Model A1000 Sanjiang bench saw (Shanghai, China). Subsequently, the specimens were surface ground and residual sawdust was removed.

### 2.3. Preparation and Performance Testing of Welded Wood

**Figure 1:**
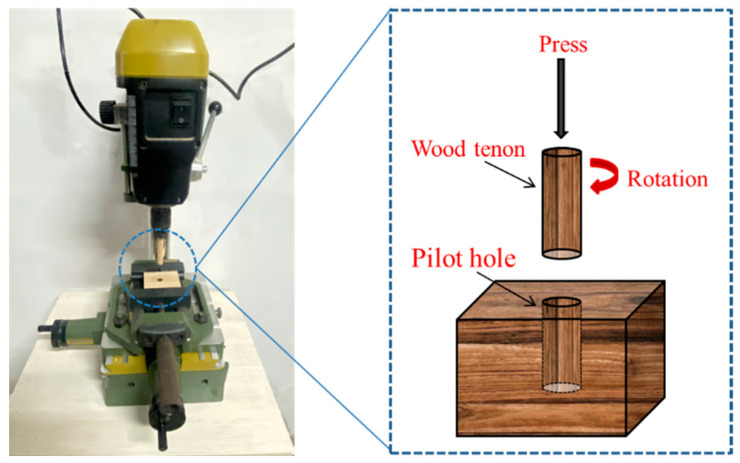
Schematic diagram of wood Rotary Welding.

The welding method is shown in Figure 1. A Proxxon Typ 2821 benchtop welding drill (Proxxon, Stuttgart, Germany) was used to fix the tenon in the center of the pilot hole and weld it at a speed of 2400 rpm. The testing method refers to the national standard GB/T 1927.21-2022 [4,19,28]. Prior to mechanical testing, all specimens were conditioned in a controlled laboratory environment at 20 ± 2 °C and 65 ± 5% relative humidity for 24 h to ensure equilibration. Tensile testing of the rotary welded joints was performed using a WDS-50KN universal testing machine (Qingdao, China) operated at a constant crosshead speed of 2.5 mm/min. Complete stress–strain curves were recorded throughout each test for comprehensive mechanical property analysis. The reported value represents the arithmetic mean of 12 independent specimens. The data were processed using Excel 2021 and Origin 2024 software, and the significance of differences was judged via the one-way analysis of variance (ANOVA) (*p* < 0.05), the error bars represent the standard deviation.

### 2.4. Characterization

The eucalyptus wood side of the tenon-eucalyptus wood welding interface was sampled. The sample was ground with KBr (1:100 mass ratio), pelletized, desiccated, and then subjected to Fourier-transform infrared (FTIR) transmittance spectroscopy. The chemistry of the weld interface was assessed by FTIR employing a Nicolet iS20 instrument (Varian, Palo Alto, CA, USA). Measurements encompassed a wavenumber range of 400–4000 cm^−1^, with a spectral resolution of 4 cm^−1^ and 32 accumulated scans. Elemental composition and bonding states were characterized via Thermo Fisher ESCALAB 250 XPS (Waltham, MA, USA) with Al Kα excitation (1486.6 eV); survey spectra utilized 100 eV pass energy (1 eV steps), while narrow scans employed 50 eV pass energy (0.05 eV steps). Crystallographic modifications were investigated on a Rigaku Ultima IV XRD system (Tokyo, Japan) using Cu Kα radiation (λ = 0.154060 nm), scanning from 5° to 90° 2θ at 5°/min with 0.02° step size under 40 kV and 120 mA. Microstructural evolution in the welded cross-section and substrate was examined using a ZEISS GeminiSEM 300 (Jena, Germany) at 12.5 kV. Prior to observation, samples were gold sputter-coated using a Quorum Q150R ES sputter coater (London, UK) to an approximate thickness of 15 nm. Thermal stability and decomposition behavior were evaluated using a Netzsch TG 209 F3 thermogravimetric analyzer (Netzsch, Rodgau, Germany). Samples weighing 6–8 mg were heated from 30 °C to 800 °C at 10 °C/min under a nitrogen atmosphere.

## 3. Results and Discussion

### 3.1. Effect of Tenon-to-Pilot Hole Diameter Ratio on the Performance of Welded Wood

**Figure 2:**
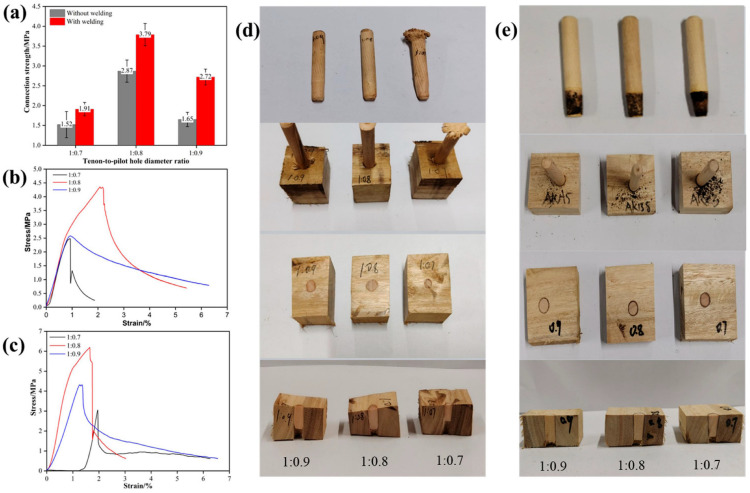
Effect of Tenon-to-pilot Hole Diameter Ratio on the Performance of Welded Wood; (**a**) connection strength, (**b**) stress–strain curves of samples without welding, (**c**) stress–strain curves welded samples, (**d**) status of samples without welding, (**e**) status of welded samples.

When Schima wood was used as the tenon, the radial section of eucalyptus wood as the substrate, the welding depth was set at 20 mm, the welding dwell time was 0 s (wooden dowel reaches the precise target depth), and the insertion angle was 90°, the effect of the tenon-to-pilot hole diameter ratio on the performance of welded wood is shown in Figure 2. As shown in Figure 2a, the tensile strength peaked at 3.79 MPa with a tenon-to-pilot hole diameter ratio of 1:0.8. Deviations to 1:0.7 or 1:0.9 reduced the strength to 2.72 MPa and 1.91 MPa, respectively. Notably, all welded specimens surpassed the strength of unwelded wood, confirming rotary welding’s effectiveness. The diameter ratio critically governs heat generation and material fusion during welding; extreme values impair thermal transfer and interfacial bonding, thereby degrading joint quality. Thus, selecting an optimal ratio is essential for enhancing welding performance. The stress–strain curves (Figure 2b,c) further reveal the effect of the tenon-to-pilot hole diameter ratio on welding performance. When the diameter ratio was 1:0.7, the stress–strain curves of the unwelded and welded wood rapidly rose to a peak and then sharply declined in the initial stage, showing typical brittle fracture characteristics, which reflected the instability of the interface bonding. When the diameter ratio was 1:0.8 and 1:0.9, the stress–strain curves showed a gradual downward trend after reaching the peak, indicating better toughness and energy absorption capacity. In particular, when the diameter ratio was 1:0.8, the stress–strain curve exhibited a higher stress peak and a longer deformation stage, indicating higher energy absorption capacity and toughness during the loading process. The reason for this phenomenon was that without welding, the connection between the tenon and the substrate mainly relied on the friction force between the tenon and the substrate, which was a relatively weak connection. This indicated that the tenon-to-pilot hole diameter ratio had an important effect on the mechanical properties of the welded connection, and both too high and too low diameter ratios were not conducive to the stability of the welded connection.

Figure 2d,e show the morphological characteristics of the tenon after welding. The conical structure of the tenon indicates that significant friction occurred during the welding process, which led to local temperature increase and softened or even melted lignin. The tenon-to-pilot hole diameter ratio had a significant effect on the morphology of the tenon after welding. When the diameter ratio was 1:0.8, the front end of the tenon presented a moderate frustum shape, indicating that the frictional heat at this time was sufficient to soften cellulose and lignin, thereby promoting the mechanical interlocking and chemical bonding of the interface materials and forming a more ideal welding interface. However, when the diameter ratio was 1:0.7, the tenon was worn excessively and presented a sharp conical shape, reflecting excessive heat accumulation leading to severe material loss and thus affecting the welding quality. Conversely, when the tenon-to-hole diameter ratio was 1:0.9, the front end of the tenon was less worn, but the interface bonding area was shallow and the welding layer thickness was insufficient, indicating that the frictional heat at this time was not sufficient to fully soften the material, resulting in limited bonding strength of the welding interface.

An appropriate tenon-to-pilot hole diameter ratio (1:0.8) could achieve a balance between frictional heat accumulation, contact area, and interface softening degree, thereby forming a more complete and uniform welding interface. When the diameter ratio was too small (1:0.9), the friction was insufficient to generate enough heat; and when the diameter ratio was too large (1:0.7), excessive friction could overheat the interface material and increase defects. This “enhancement-attenuation” dynamic balance helps to explain the balance observed in parameter optimization for rotary welding technology. The results of this study are consistent with the conclusions of Kanazawa et al. [29] on European hardwoods, that is, an appropriate diameter ratio is the key to enhancing welding strength. However, the results of this study on eucalyptus wood further indicate that its higher density and uniform fiber arrangement provide advantages in optimizing the diameter ratio. These structural features likely promote more effective heat transfer and material bonding during welding.

### 3.2. Effect of Welding Dwell Time on the Performance of Welded Wood

**Figure 3:**
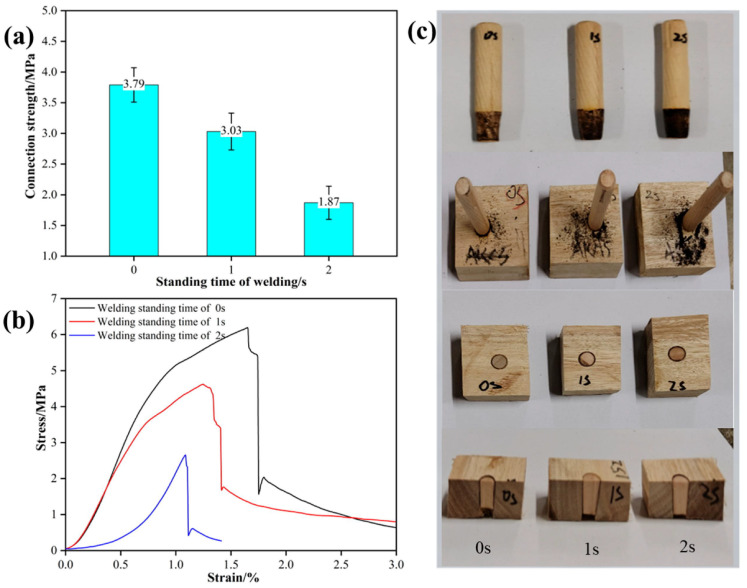
Effect of welding standing time on the performance of welded wood; (**a**) connection strength, (**b**) stress–strain curves, (**c**) status of welded wood. Note: Dwell time 0 s—wooden dowel reaches the precise target depth; dwell time 1 s, 2 s—the continuous welding time at the target depth.

When Schima wood was used as the tenon, the radial section of eucalyptus wood as the substrate, the tenon-to-pilot hole diameter ratio was 1:0.8, the welding depth was set at 20 mm, and the insertion angle was 90°, the effect of welding dwell time on the performance of welded wood is shown in Figure 3. Figure 3a shows that the welding dwell time has a significant effect on the pull-out strength of welded eucalyptus wood. When the dwell time was 0 s, the pull-out strength reached the highest value of 3.79 MPa; whereas when the dwell time was extended to 1 s and 2 s, the pull-out strength decreased to 3.03 MPa and 1.87 MPa, respectively. These results indicated that a short welding time was sufficient to achieve a strong mechanical interlock, while a longer welding time may cause thermal damage or degradation, reducing the effective mechanical interlocking between the tenon and the hole wall, thereby decreasing the connection strength. This is consistent with the findings of Belleville et al. [30], who discovered that excessive welding time could lead to a decrease in connection strength due to thermal degradation and carbonization. This study further confirms the effect of welding dwell time on the mechanical properties of the welded interface of eucalyptus wood, demonstrating that appropriately controlling the welding dwell time is crucial for maintaining efficient connections.

The stress–strain curves (Figure 3b) clearly reveal the effect of welding dwell time on the loading behavior of the welded joint. The results show that the specimen with a dwell time of 0 s exhibited the highest stress peak (4.5 MPa) and the longest deformation stage, indicating that both the interface bonding strength and toughness were at optimal levels. However, as the welding dwell time was extended to 2 s, the stress peak significantly decreased, and the fracture mode gradually shifted from ductile to brittle. This transition is closely related to the thermal degradation of wood fibers and excessive carbonization of the interface, indicating that an extended dwell time may lead to material degradation, thereby causing the fracture of wood fibers. The morphological characteristics of the tenon after welding (Figure 3c) reveal that dwell time significantly affects interfacial carbonization. At 0 s, only slight carbonization occurred with complete, uniform bonding. At 1 s, carbonization increased with localized material loss, while at 2 s, severe carbonization created significant wear and interfacial unevenness. This progressive carbonization weakens mechanical interlocking and increases defects, reducing connection strength. The underlying mechanism involves heat accumulation from friction, causing thermal degradation of wood components. When the dwell time is short, generated heat sufficiently softens and rearranges lignin and cellulose, forming tight mechanical interlocks and chemical bonds. However, extended dwell times cause excessive heat accumulation, leading to pronounced degradation of hemicellulose—the most thermally sensitive component—and cellulose, which releases volatile substances and generates excessive carbonized products. This transition, dominated by hemicellulose degradation, explains the shift from optimal bonding to weakened interface strength. The results of this study verify the effect of welding dwell time on the interface properties of rotary welded eucalyptus wood and further reveal the coupling relationship between welding thermal effects and interface properties. Compared with the study on linear vibration welding by Pizzi [31], this study further confirms the dynamic balance relationship between heat accumulation and material degradation in rotary welding. Controlling the welding dwell time between 0 s and 1 s can achieve the optimal connection strength and interface stability for eucalyptus wood welding. In addition, the evolution of tenon carbonization morphology also provides new empirical support for optimizing welding time.

### 3.3. Effect of Welding Depth on the Performance of Welded Wood

**Figure 4:**
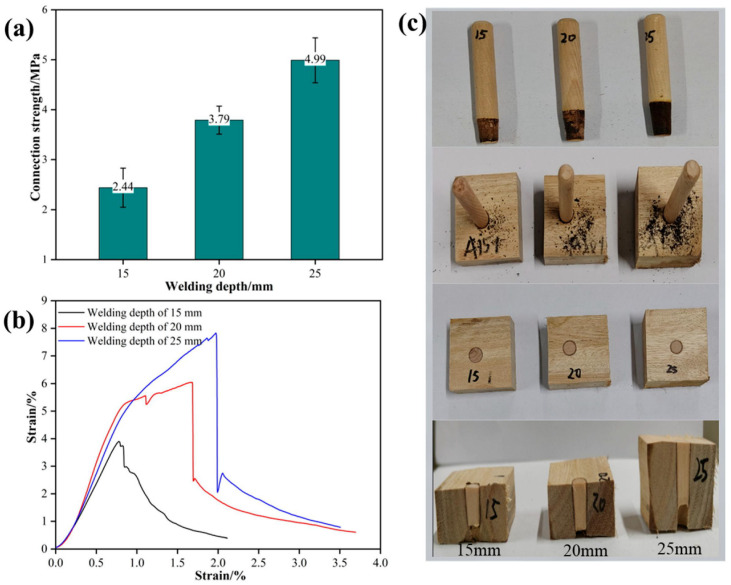
Effect of welding depth on the performance of welded wood; (**a**) connection strength, (**b**) stress–strain curves, (**c**) status of welded wood.

Welding depth is also one of the key process parameters that affect the performance of rotary welding. When Schima wood was used as the tenon, the radial section of eucalyptus wood as the substrate, the tenon-to-pilot hole diameter ratio was 1:0.8, the welding dwell time was 0 s, and the insertion angle was 90°, the effect of welding depth on the performance of welded wood is shown in Figure 4. As shown in Figure 4a, when the welding depth was 25 mm, the tensile strength reached the maximum value of 4.99 MPa; when the welding depth was 20 mm, the tensile strength was 3.79 MPa; and when the welding depth was 15 mm, the tensile strength was the lowest, at only 2.44 MPa. As the welding depth increased, the tensile strength gradually improved. This phenomenon can be attributed to the increased welding depth providing a larger interface bonding area and more uniform heat distribution, thereby enhancing the mechanical interlocking and chemical bonding strength of the interface. Cornuault et al. [10] found that deeper welding helps to increase the connection strength of medium-density fiberboard. This study further points out that in higher-density eucalyptus wood, the advantages of deep welding are more pronounced, indicating that welding depth plays a key role in the high-density structure of eucalyptus wood.

As shown in Figure 4b, the stress–strain curves further reveal the effect of welding depth on mechanical properties. In the specimens with a welding depth of 25 mm, the stress peak was the highest and the deformation stage was the most durable, indicating higher toughness and energy absorption capacity. In contrast, the specimens with a welding depth of 15 mm showed obvious brittle fracture characteristics during loading, indicating that a shallower welding depth fails to form sufficient bonding force at the interface. Deeper welding depths, on the other hand, can better disperse internal stresses, thereby enhancing the stability of the connection.

The welding depth has a significant effect on the degree of carbonization and morphological characteristics at the front end of the tenon (Figure 4c). When the welding depth reached 25 mm, the front end of the tenon was significantly carbonized and presented a smooth conical shape. This indicates that a larger welding depth increases the accumulation of frictional heat, promoting the thermal degradation of cellulose and hemicellulose in the wood. Although the degree of carbonization is high, the interface bonding area remains tight, indicating that moderate carbonization can enhance the mechanical interlocking effect. Therefore, appropriately increasing the welding depth not only enhances the connection strength but also improves the interface stability, providing more reliable technical support for wooden structural buildings and furniture manufacturing. The results of this study further refine the research on welding depth in different woods by Župčić et al. [32], clarifying the optimization range of welding depth in high-density eucalyptus wood and providing a new theoretical basis for the selection of rotary welding process parameters.

### 3.4. Effect of Welding Base Surface on the Performance of Welded Wood

**Figure 5:**
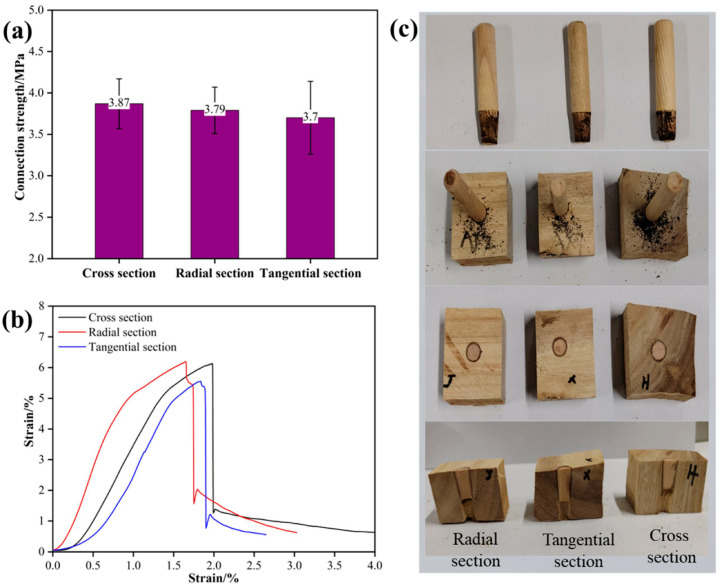
Effect of welding surface on the performance of welded wood; (**a**) connection strength, (**b**) stress–strain curves, (**c**) status of welded wood.

When Schima wood was used as the tenon, the tenon-to-pilot hole diameter ratio was 1:0.8, the welding depth was 20 mm, the welding dwell time was 0 s, and the insertion angle was 90°, the effect of the welding base surface on the performance of welded wood is shown in Figure 5. As shown in Figure 5a, the connection strength was highest (3.87 MPa) when the radial section was used as the welding base surface, followed by the tangential section (3.79 MPa), and the lowest (3.70 MPa) when the cross-section was used. This indicates that in the welding process of eucalyptus wood, although the choice of welding base surface has a certain effect on the connection strength, the differences among the three are relatively small, which may reflect the relatively uniform fiber response of eucalyptus under welding conditions. The stress–strain curves in Figure 5b reveal subtle differences in the mechanical behavior after welding on different base surfaces. Specimens welded on the radial section maintained a longer time in the high-stress area, showing higher tensile strength and ductility. This may be due to the advantages of the radial section in thermal energy transfer efficiency and fiber orientation, which makes the welding interface more resistant to fracture when subjected to force.

After comparative analysis of the morphological characteristics of the tenon after welding on the three base surfaces (Figure 5c), it was found that when the radial section was used as the welding base surface, the welding part of the tenon was neater, with fewer signs of charring and thermal damage. In contrast, the cross-section and tangential section showed more irregular heat-affected areas, which may be caused by the fiber orientation and uneven heat distribution. The fiber direction of wood is closely related to the heat propagation path, which in turn affects the welding effect and mechanical properties. Therefore, the heat-affected areas of the cross-section and tangential section are relatively small. This study confirms that in eucalyptus wood welding, although the choice of welding base surface has a certain effect on welding performance, the difference is relatively small.

### 3.5. Effect of Insertion Angle on the Performance of Welded Wood

**Figure 6:**
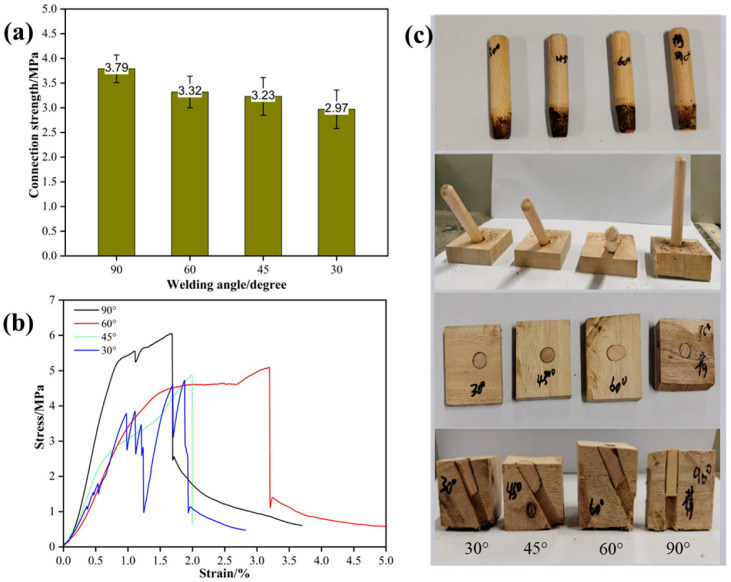
Effect of Insertion Angle on Wood Welding Performance From left to right: 30°, 45°, 60°, 90°; (**a**) connection strength, (**b**) stress–strain curves, (**c**) status of welded wood.

When Schima wood was used as the tenon, the radial section of eucalyptus wood as the substrate, the tenon-to-pilot hole diameter ratio was 1:0.8, the welding depth was set at 20 mm, and the welding dwell time was 0 s, the effect of insertion angle on the performance of welded wood is shown in Figure 6. As shown in Figure 6a, the connection strength was highest at a angle of 90°, reaching 3.79 MPa. As the angle decreased, the connection strength gradually decreased: it was 3.32 MPa at 60°, 3.23 MPa at 45°, and the lowest at 30°, only 2.97 MPa. The stress–strain curves in Figure 6b further reveal the effect of different insertion angles on the mechanical response of welded wood. Specimens welded at a 90° angle exhibited higher peak stress and more ductile strain behavior, indicating better mechanical stability and toughness of the welded joint at this angle. In contrast, specimens welded at a 30° angle failed rapidly after reaching peak stress, indicating poorer mechanical properties at lower angles. This phenomenon may be related to the reduced contact area and uneven thermal effects. The effect of different welding angles on welding was further confirmed in the morphological characteristics of the wood after welding (Figure 6c). Specimens welded at a 90° angle showed less thermal damage and a more uniform welding surface; whereas specimens welded at a 30° angle exhibited obvious charring and uneven welding zones, which may affect the overall strength and stability of the joint.

Insertion angle critically modulates heat distribution during welding, thereby controlling thermal decomposition at the tenon-substrate interface. This study’s systematic examination of eucalyptus wood revealed that larger angles consistently enhanced mechanical properties and joint stability. These results demonstrate that optimizing angular orientation is fundamental for achieving robust, high-quality welds in practical manufacturing.

### 3.6. Effect of Tenon Species on the Performance of Welded Wood

**Figure 7:**
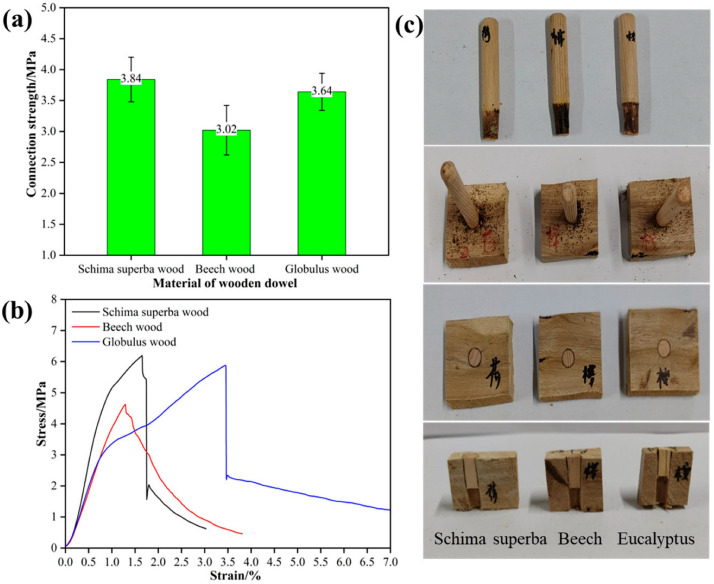
Effect of Tenon Species on the Performance of Welded Wood; (**a**) connection strength, (**b**) stress–strain curves, (**c**) status of welded wood.

When the radial section of eucalyptus wood was used as the substrate, the tenon-to-pilot hole diameter ratio was 1:0.8, the welding depth was set at 20 mm, the welding dwell time was 0 s, and the insertion angle was 90°, the effect of tenon species on the performance of welded wood is shown in Figure 7. As shown in Figure 7a, Schima tenons exhibited the highest connection strength of 3.84 MPa; eucalyptus tenons were next, at 3.64 MPa; while Zelkova tenons had a relatively lower connection strength, only 3.02 MPa. Despite eucalyptus having a higher density than Schima, the welding strength of Schima tenons was higher than that of eucalyptus tenons when eucalyptus was used as the substrate. This result indicates that welding strength is not only affected by wood density but is also closely related to fiber orientation, as suggested by the neater surfaces and fewer signs of thermal damage observed in Schima tenons. On the one hand, the uniformity and directional consistency of fibers have an important influence on the overall performance of the welding area. The arrangement of Schima fibers may be more conducive to forming a strong welding interface than that of eucalyptus. On the other hand, different woods generate different internal stresses during the welding, which in turn affect the strength of the welded wood. Schima may generate smaller internal stresses during welding, making the welding point more stable and reliable. In contrast, eucalyptus may experience higher stress concentration during welding due to its higher density, which paradoxically reduces the welding strength.

As shown in Figure 7b, the stress–strain curves reveal the differences in behavior of different tenon materials under load. The curve of Schima tenon is steeper, indicating that it can withstand higher stress in the initial stage, showing higher initial stiffness and strength. In contrast, the curve of Zelkova tenon is gentler, showing lower stress under the same strain. This difference may be related to the lower density of Zelkova and its unique fiber arrangement, which may produce a relatively weaker mechanical response under load. From the morphological characteristics of the tenon after welding (Figure 7c), it can be observed that the surfaces of Schima and eucalyptus tenons are neater after welding, showing fewer signs of thermal damage; while Zelkova tenon shows more charring and irregular welding interfaces. These observations are consistent with the results of connection strength, further illustrating the impact of tenon species on welding quality and also indicating that the compatibility between the tenon and the substrate affects the structural integrity of the welding interface and the connection strength of the welded wood.

### 3.7. FT-IR Spectroscopy Analysis

**Figure 8:**
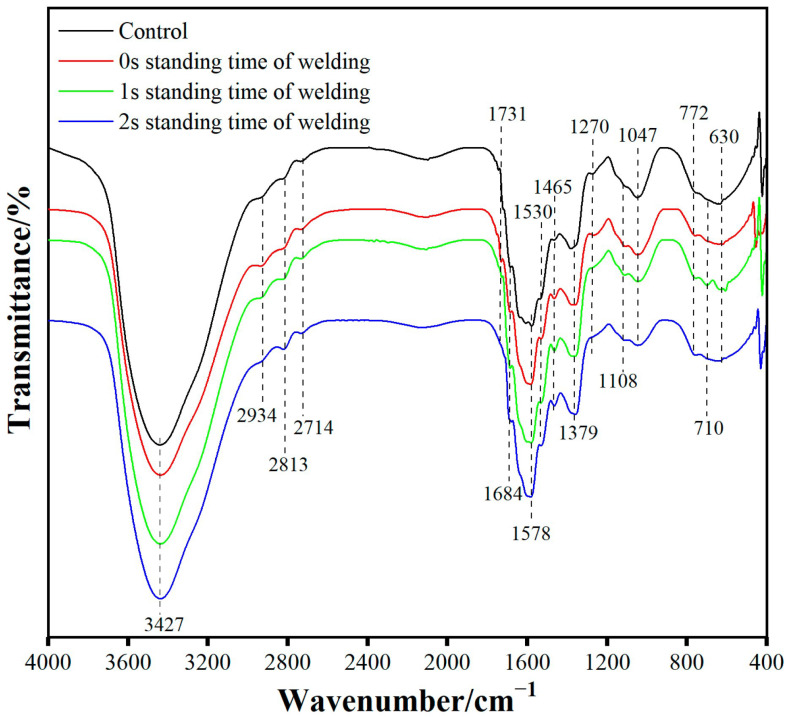
FT-IR results of eucalyptus wood substrates at different welding dwell times.

Figure 8 shows the FT-IR results of eucalyptus wood substrates at different welding dwell times. The stretching vibration of the bonded OH groups or water were detected at 3427 cm^−1^; the stretching vibration of C-H was observed between 2714 and 2934 cm^−1^; the stretching vibration of C=O was identified at 1578, 1684 and 1731 cm^−1^, while C-H bending vibrations in methyl and methylene groups were noted at 1465 and 1379 cm^−1^. The methoxy (-OCH_3_) stretching vibration at 1270 cm^−1^ indicated the syringyl and guaiacyl structures of lignin. The stretching vibrations of the polysaccharide carbon C-O-C and C-OH bonds in hemicellulose were found at 1108 cm^−1^, while the vibration at 1047 cm^−1^ originated from the polysaccharide carbon in hemicellulose. The bending vibrations with the C-O and C-C bonds as well as out of plane bending vibrations with OH groups were recorded between 630 and 772 cm^−1^ [19,20].

As the welding dwell time increased, the intensity of the stretching vibration absorption peak at 3427 cm^−1^ gradually decreased. This indicated that during the welding process, hemicellulose underwent degradation, and the hydroxyl groups in the amorphous regions of cellulose dehydrated to form ether bonds, resulting in a reduction in the number of hydroxyl groups. At 2813 cm^−1^, the absorption peak intensity of the unwelded samples and those with welding dwell times of 0 s and 1 s decreased, while the absorption peak intensity increased for samples with a welding dwell time of 2 s. This suggested that as the welding dwell time extended, the organic components in the wood (including cellulose) might undergo localized carbonization. During the carbonization process, some complex organic molecules underwent thermal decomposition, forming carbon-containing compounds. The thermal instability of hemicellulose was manifested by the weakening of the absorption peaks at 1047 and 1108 cm^−1^ as the welding dwell time increased.

The peak at a wavenumber of 1578 cm^−1^ increased in intensity with the increase in welding dwell time. This was because, as the welding dwell time extended, the surface and interior of the eucalyptus wood substrate experienced prolonged high temperature and friction, leading to more intense oxidation reactions. The components in wood, especially cellulose, hemicellulose, and lignin, produced carbonyl-containing compounds such as esters, aldehydes, and carboxylic acids during the oxidation process. As the oxidation reaction deepened, the C=O absorption peak became stronger. A new absorption peak appeared at 1731 cm^−1^, which is usually associated with the stretching vibration of C=O, indicating that hemicellulose is prone to degradation under high-temperature conditions, and the C=O formed after degradation generates a new absorption peak at 1731 cm^−1^. The infrared spectrum further showed that the welded wood had the least fluctuation in the range of 1270–1578 and 630–772 cm^−1^, indicating that lignin has relative stability during the welding process and its structure did not show obvious degradation.

### 3.8. XPS Analysis

**Figure 9:**
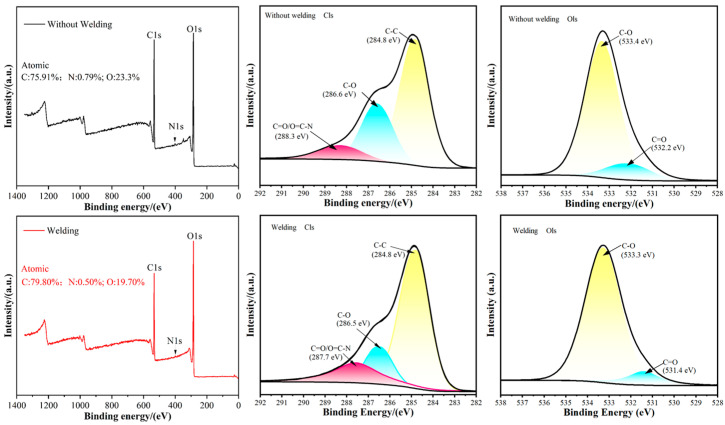
XPS Results of Unwelded and Welded Materials.

XPS analysis of unwelded and welded eucalyptus wood substrates (Figure 9) revealed shifts in elemental composition, with carbon content marginally increasing while oxygen and nitrogen decreased. These alterations suggest welding’s high-temperature and high-pressure environment modified surface chemistry and likely facilitated new chemical bond formation.

High-resolution C1s and O1s spectra demonstrated that welding modified the chemical states of wood surfaces. Specifically, C1s spectra showed distinct variations in carbon bonding environments between unwelded and welded samples, indicating welding induced changes in surface carbon compound chemistry—including reduced C-O bonds and elevated C=O bonds. These transformations probably stem from thermal decomposition of lignin and cellulose during welding [19].

Meanwhile, O1s spectra exhibited enhanced C-O bond content post-welding compared to unwelded specimens. Consequently, welding’s high-temperature, high-pressure conditions drove surface chemical composition changes encompassing structural reorganization and novel bond formation. These findings underscore the significant influence of welding parameters on wood’s chemical properties.

### 3.9. XRD Analysis

**Figure 10:**
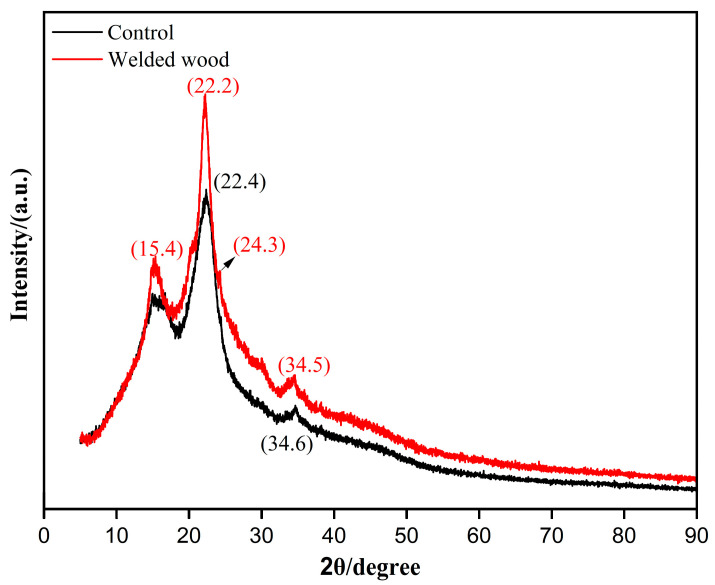
XRD Results of Eucalyptus Before and After Welding.

XRD patterns of original and welded eucalyptus wood substrates (Figure 10) revealed pronounced peaks at 15.4° and 22.2°, representing intensified diffraction from specific crystallographic planes. This enhancement indicates that welding promoted development of these crystal surfaces [19]. The thermal cycle during welding modified these peak intensities, suggesting that welding heat likely induced reorganization of amorphous cellulose regions into a more organized crystalline arrangement. Consequently, welding alters the microstructural organization in the weld zone, driving the system toward enhanced crystalline order. This orderly arrangement structure is similar to the ideal structure in metallurgy, such as bainite or martensite structure [33,34]. These structures are highly valued for their excellent mechanical properties, including high strength, good toughness and ductility. In eucalyptus wood welding materials, this orderly crystal structure significantly improves its comprehensive mechanical properties, covering yield strength, tensile strength and ductility.

### 3.10. SEM Analysis

**Figure 11:**
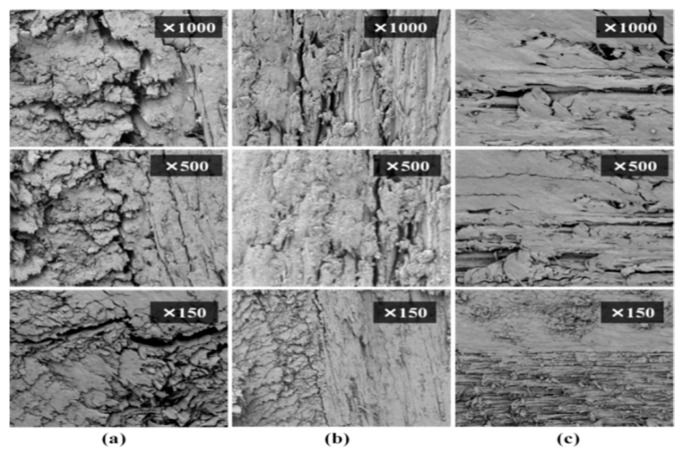
SEM images of the cross-sections of the tenons. (**a**) the control group; (**b**) welding dwell times of 0 s; (**c**) welding dwell times of 2 s.

Figure 11 shows the SEM images of the cross-sections of the connections of unwelded and welded eucalyptus wood substrates. At a magnification of 1000 times, the unwelded wood exhibits a rough surface with a relatively loose fiber structure, and there are obvious pores and cracks. At a magnification of 500 times, the surface cracks become clearer. At a magnification of 150 times, the overall surface condition and the degree of cracking are more evident. For the wood with a welding dwell time of 0 s, at a magnification of 1000 times, the microstructure begins to change, the surface becomes smoother, but there are still small gaps and melting areas. At a magnification of 500 times, the surface gaps decrease, becoming more uniform, and the melting area increases. At a magnification of 150 times, the surface is smoother and tighter, showing the subtle changes caused by a short welding time. When the welding dwell time is 2 s, at a magnification of 1000 times, the surface cracks are significantly reduced, showing obvious melt pools and solidification structures. At a magnification of 5000 times, a large number of solidification structures can be seen, and the surface is smoother and more uniform. At a magnification of 150 times, the cracks disappear, the surface is tight and smooth, and the difference between the welded and unwelded areas is clear.

**Figure 12:**
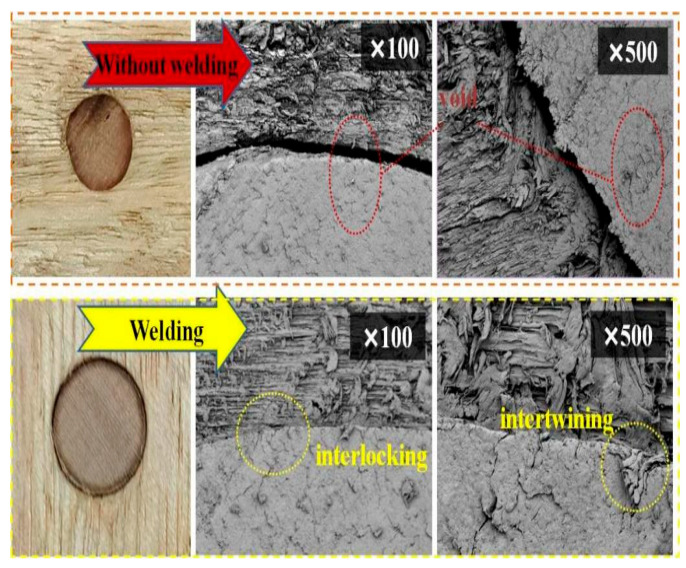
SEM images (Top View) of the cross-sections of the tenons, with the control group and dwell times of 0 s from top to bottom.

From the top view of the connection interface (Figure 12), the cracks in the unwelded wood are clearly visible, and no material mixing or morphological changes are observed. At a magnification of 100 times, the gaps between the tenon and the substrate are further highlighted. In contrast, in the welded wood, the interface becomes indistinct, and the degree of material fusion increases. At a magnification of 500 times, the wood fibers are closely connected, and at a magnification of 100 times, the mechanical interlocking between fibers is further highlighted. During the welding process, the high temperature melts the fiber surface and embeds it, increasing the tightness of the interface. This process forms a complex structure at the interface, enhancing mechanical interlocking and improving bonding strength. At the same time, the high temperature softens and partially decomposes the fibers, and the released components form new chemical bonds, further enhancing adhesion. The applied pressure also promotes close contact and interlocking between fibers. When the material cools, it solidifies to form a stable welded joint.

In summary, in the early stages of tenon welding, the microstructure begins to change. Small melt pools cool and solidify rapidly, effectively repairing existing micro-defects. However, a long welding process can lead to excessive heat accumulation and an increase in melt pool size, thereby changing the microstructure of the wood and potentially having a negative impact on the wood’s crystal structure. These results clearly show that there is a crucial balance between welding time and structural integrity. This balance is extremely important for optimizing welding process parameters and improving the overall performance of wood connections.

### 3.11. TG/DTG Analysis

**Figure 13:**
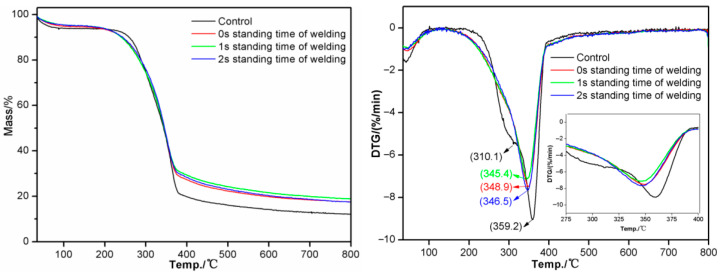
TG/DTG Results of weld wood at different welding dwell times.

Figure 13 shows the TG/DTG test results of eucalyptus wood substrates under different welding dwell times. Wood is mainly composed of cellulose, hemicellulose, lignin, and a small amount of extractives. Under high temperatures, hemicellulose is the least stable, first thermally degrading into polysaccharides and then forming furan compounds, while the aromatic ring structure of lignin is relatively stable [35]. The TG curves show that the weight loss process of all samples can be divided into three stages [36,37,38]. The first stage: Weight loss is relatively gentle, mainly due to the evaporation of moisture in the wood and the thermal degradation of some extractives. The second stage: Weight loss is more intense, mainly involving the thermal degradation of cellulose and hemicellulose, with products being mostly volatile substances. The third stage: Weight loss continues slowly, possibly due to the thermal degradation of a small amount of lignin and further thermal degradation of extractives triggered by higher temperatures. Finally, lignin forms solid compounds through high aromatization and carbonization.

Both unwelded and welded wood samples show similar weight loss trends, but the residual rate of the welded samples is higher, indicating a relative increase in lignin content at the welding interface. This may be because during the welding process, the thermal degradation of hemicellulose and cellulose leads to a relative increase in lignin content. In the second stage, the thermal degradation of hemicellulose and cellulose causes differences between unwelded and welded samples, which may be due to the thermal decomposition, reconfiguration, and partial carbonization of cellulose and hemicellulose during the welding process. The increase in weight loss rate in the third stage may be due to the thermal degradation of a small amount of lignin. These results indicate that the welding process not only changes the chemical composition of wood but also affects its pyrolysis behavior.

The DTG curves show that the maximum decomposition rate of the unwelded sample occurs at 359.2 °C. In contrast, the samples with welding dwell times of 0 s, 1 s, and 2 s have maximum decomposition rates at 348.9 °C, 345.4 °C, and 346.5 °C, respectively. This indicates that as the welding time increases, the thermal degradation of cellulose during the welding process increases, the long chains in the crystalline regions break into shorter chains, leading to a decrease in the temperature of the maximum rate of pyrolysis, which is consistent with the research results of Zhu et al. [18]. Moreover, the thermal degradation of hemicellulose and cellulose during the welding process leads to a relative increase in lignin content, thereby increasing the pyrolysis rate. At the same time, the friction during the welding process may cause more lignin to melt into the interface material, further increasing the pyrolysis rate.

## 4. Conclusions

1. This study successfully achieved its objective to systematically evaluate rotary welding parameters for eucalyptus wood and characterize their effects on joint performance. The central results demonstrate clear optimization pathways: the tenon-to-pilot hole diameter ratio of 1:0.8 maximized connection strength at 3.79 MPa, zero-second dwell time proved optimal as longer durations caused thermal degradation reducing strength by over 50%, and increasing welding depth to 25 mm significantly enhanced performance compared to shallower depths.
2. Chemical and microstructural analyses revealed that welding-induced partial degradation of hemicellulose and cellulose led to new chemical bond formation and increased carbonyl compounds. XRD showed increased wood crystallinity, SEM confirmed tighter interfaces with enhanced mechanical interlocking, and TGA verified improved thermal stability. These integrated modifications collectively explain the mechanical performance improvements.
3. Several limitations should be noted. The study examined only eucalyptus as substrate wood, limiting generalization to other species. Only selected process parameters were evaluated, leaving additional factors unexplored. Furthermore, all testing was conducted under controlled laboratory conditions, which may not fully replicate industrial manufacturing or long-term field performance.
4. Future research directions should include testing rotary welding on other wood species to validate parameter transferability, and evaluating joint performance under varying humidity conditions to assess moisture resistance. Additionally, testing under real-world usage loads would verify durability in practical structural applications.
5. Despite these limitations, the findings provide practical guidance for optimizing rotary welding protocols in sustainable wood manufacturing.

## Data Availability

The original contributions presented in this study are included in the article. Further inquiries can be directed to the corresponding authors.
